# A Phase II study of avenciguat, a novel soluble guanylate cyclase activator, in patients with systemic sclerosis: Study design and rationale of the VITALISScE™ study

**DOI:** 10.1177/23971983241291923

**Published:** 2024-11-07

**Authors:** Dinesh Khanna, Jeska de Vries-Bouwstra, Anna-Maria Hoffmann-Vold, Masataka Kuwana, Andrea Hsiu Ling Low, Susanna Proudman, Mary Flack, Anjli Kukreja, Nora Fagan, Oliver Distler

**Affiliations:** 1 Department of Internal Medicine, University of Michigan Scleroderma Clinic, Ann Arbor, MI, USA; 2 Department of Rheumatology, Leiden University Medical Center, Leiden, The Netherlands; 3 Department of Rheumatology, Oslo University Hospital, Oslo, Norway; 4 Department of Rheumatology, University Hospital Zurich, University of Zurich, Zurich, Switzerland; 5 Department of Allergy and Rheumatology, Nippon Medical School Graduate School of Medicine, Tokyo, Japan; 6 Department of Rheumatology and Immunology, Singapore General Hospital, Singapore; 7 Duke-National University of Singapore Medical School, Singapore; 8 Discipline of Medicine, University of Adelaide and Rheumatology Unit, Royal Adelaide Hospital, Adelaide, SA, Australia; 9 TA Inflammation Medicine, Boehringer Ingelheim Pharmaceuticals, Inc., Ridgefield, CT, USA; 10 Translational Medicine & Clinical Pharmacology, Boehringer Ingelheim Pharmaceuticals, Inc., Ridgefield, CT, USA; 11 Global Biostatistics & Data Sciences, Boehringer Ingelheim Pharmaceuticals, Inc., Ridgefield, CT, USA

**Keywords:** SSc, clinical trial, sGC activator, microvasculopathy, fibrosis

## Abstract

**Introduction:**

Systemic sclerosis is a rare autoimmune connective tissue disease characterised by (1) microvasculopathy; (2) immune dysregulation; and (3) progressive fibrosis of the skin and internal organs. Soluble guanylate cyclase plays an important role in maintaining vascular and immunological homeostasis and preventing organ fibrosis. Pharmacological modulation of soluble guanylate cyclase with soluble guanylate cyclase stimulators has shown anti-inflammatory and antifibrotic effects in animal models of systemic sclerosis, with a trend towards clinical efficacy in a Phase II study (RISE-SSc). However, the efficacy of soluble guanylate cyclase stimulators may be reduced under conditions of hypoxia and oxidative stress. Soluble guanylate cyclase activators have the potential to overcome this limitation. This paper describes the study design of VITALISScE™, a Phase II clinical trial assessing the efficacy, safety and tolerability of avenciguat, a novel soluble guanylate cyclase activator in patients with active systemic sclerosis at risk of progression.

**Methods:**

The VITALISScE™ study (NCT05559580) is evaluating the action of avenciguat on all three aspects of systemic sclerosis pathophysiology. The primary endpoint is the rate of decline in forced vital capacity (mL) over 48 weeks. Secondary endpoints include absolute change from baseline at Week 48 in modified Rodnan skin score, Health Assessment Questionnaire Disability Index score and the proportion of responders based on the revised Composite Response Index in Systemic Sclerosis. Additional endpoints include a composite assessment of Raynaud’s phenomenon, digital ulcer burden, functional outcomes and quality of life, safety, pharmacokinetics, and biomarkers associated with systemic sclerosis and the mechanism of action of avenciguat.

**Results:**

VITALISScE™ is an ongoing, multicentre (180 sites; 38 countries), placebo-controlled, double-blind, parallel-group, Phase II clinical study. Recruitment is currently ongoing.

**Conclusions:**

The VITALISScE™ study is assessing the efficacy, safety and tolerability of avenciguat in patients with active systemic sclerosis at risk of progression. Results will inform further development of avenciguat.

**Trial Registration:**

VITALISScE™; EU CT No. 2022-500332-11-00; Clinicaltrials.gov: NCT05559580 (https://www.clinicaltrials.gov/study/NCT05559580).

## Plain Language Summary

Systemic sclerosis, or SSc, is a rare autoimmune disease where a person’s immune system attacks their body and causes thickening or scarring, known as fibrosis. Fibrosis can happen in the skin and in other tissues, such as the lungs, and may cause bothersome symptoms throughout the body. Alongside fibrosis, patients with SSc may also have damage to their blood vessels and problems with their immune system. Some treatments for SSc work by targeting an enzyme in the blood called soluble guanylate cyclase, or sGC for short. These treatments either stimulate or activate sGC, which helps to maintain normal blood vessel and immune system activity. Effects of the sGC pathway can also help to prevent fibrosis. The activity of sGC stimulators may decrease when there are low oxygen levels in tissues affected by fibrosis. sGC activators have been suggested to work well in low-oxygen environments and may be useful treatments for patients with SSc. The VITALISScE™ study is looking at an sGC activator called avenciguat in patients with SSc. This study is currently ongoing across multiple countries. It includes patients with SSc at risk of their disease getting worse. During the study, patients will randomly receive either avenciguat or a placebo (non-active drug). Patients can take some of their usual medications for SSc, with some restrictions. The impact on lung function will be measured over 48 weeks. Through the study, the investigators will look at changes in participants’ lung function, skin thickness, ability to carry out daily activities, quality of life and any other effects of the medicine. Researchers want to understand if it is possible to reduce fibrosis by targeting the sGC pathway.

## Introduction

Systemic sclerosis (SSc) is a rare, heterogeneous, autoimmune connective tissue disease^1,2^ characterised by (1) microvasculopathy, (2) immune dysregulation and (3) progressive fibrosis of the skin and internal organs.^3–6^ The pathogenesis of SSc involves a complex interplay between these three processes,^1,2^ with microvascular injury and immune dysregulation leading to the release of pro-inflammatory cytokines from macrophages and lymphocytes^7,8^ and fibroblast activation in multiple organ systems.^3^ By the time a patient is diagnosed, microvasculopathy, immune dysregulation and fibroblast activation are occurring simultaneously, leading to disease progression with considerable morbidity and mortality.^9^ The leading cause of death in SSc is lung fibrosis,^10^ but involvement of the skin, joints, gastrointestinal tract and cardiovascular system can also cause significant disruption to patients’ lives.^11,12^

**Figure 1.**
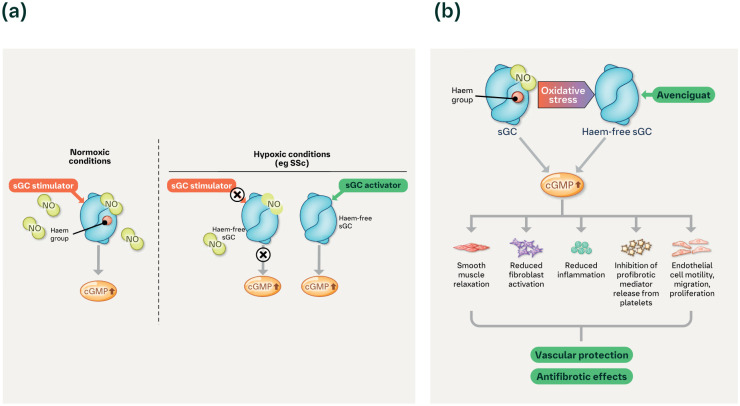
sGC stimulators and activators mode of action in SSc. (a) sGC stimulators operate efficiently under normoxic conditions whereas sGC activators are able to operate efficiently under conditions of oxidative stress, as seen in patients with SSc. (b) Increased cGMP mediates multiple downstream effects involved in vascular protection and antifibrotic effects. cGMP: cyclic guanosine monophosphate; NO: nitric oxide; sGC: soluble guanylate cyclase; SSc: systemic sclerosis.

The soluble guanylate cyclase (sGC) pathway is critical to maintaining vascular homeostasis, regulating the immune system and preventing organ fibrosis. sGC, when bound to nitric oxide (NO) and haem, activates the sGC–cyclic guanosine monophosphate (cGMP) pathway and stimulates cGMP production.^13^ This, in turn, plays a key role in regulating aspects of the underlying pathophysiology of SSc, such as smooth muscle relaxation,^14^ fibrosis,^15^ platelet activity^16^ and endothelial cell function.^17^ In the fibrotic tissues of patients with SSc, hypoxia and oxidative stress often occur^12^ due to deposition of extracellular matrix, luminal narrowing, impaired angiogenesis and reduction in the number of capillaries, and vasoconstriction.^7^ This leads to lower levels of NO and dissociation of the haem moiety that impairs the activity of sGC (Figure 1(a)).^13,18,19^

Restoring sGC activity in a hypoxic environment is a potential new approach to the treatment of SSc.^11,20^ sGC stimulation dose-dependently inhibits collagen release in dermal fibroblasts from patients with SSc,^21^ by blocking transforming growth factor β (TGF-β)^22–24^ and inhibiting TGF-β1-induced extracellular signal-regulated kinase phosphorylation.^25^ In addition, it promotes angiogenesis^26^ and has anti-inflammatory activity.^27^ However, as sGC stimulators rely on haem-bound NO to modulate the sGC–cGMP pathway, their efficacy may be reduced under conditions of hypoxia and oxidative stress.^19,28^ In contrast, sGC activators offer conceptual benefits over sGC stimulators as they function independently of haem and NO through direct binding to the haem site, stabilising sGC in an active form even in environments of hypoxia and oxidative stress.^19,24^

Avenciguat (BI 685509) is an sGC activator that has been shown to function under conditions of persistent oxidative stress (Figure 1(b)).^19^ Preclinical data in mouse models of SSc have shown that avenciguat significantly reduces bleomycin-induced skin and lung fibrosis in a dose-dependent manner and also reduces the levels of cytokines and chemokines that contribute to microvasculopathy, immune dysregulation and fibrosis. Avenciguat may therefore offer disease-modifying potential for all three aspects of the underlying pathophysiology of SSc.^24^

Previous clinical studies in healthy volunteers and patients with diabetic kidney disease and liver cirrhosis have shown that avenciguat has an acceptable pharmacokinetic, safety and tolerability profile.^29,30^ Avenciguat displays linear pharmacokinetics^29^ and lowers albuminuria in patients with chronic kidney disease,^31^ potentially due to microvascular effects in the glomeruli. Based on these data from studies in other indications, a positive risk–benefit profile was suggested for avenciguat, paving the way for the Phase II VITALISScE™ study in patients with SSc.

This manuscript describes the study design of VITALISScE™ (NCT05559580), a Phase II, placebo-controlled study of avenciguat that is being conducted in patients with active SSc at risk of progression to assess its efficacy, safety and tolerability in conjunction with standard-of-care therapy.

## Methods

### VITALISScE™ study design

**Figure 2.**
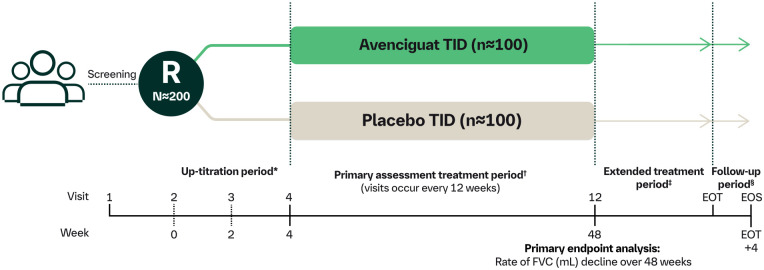
VITALISScE™ study design. EOS: end of study; EOT: end of treatment; FVC: forced vital capacity; R: randomisation; TID: three times daily. *Avenciguat will be up-titrated as tolerated, from a low dose TID to a medium dose TID after 2 weeks, and then to the maximum dose TID after an additional 2 weeks. ^†^Maximum or highest tolerated dose TID. ^‡^After completing 48 weeks of treatment, patients may continue to receive their assigned study treatment in the extended treatment period, until the last patient has completed the primary assessment treatment period. ^§^The follow-up period is 4 weeks.

The Phase II VITALISScE™ study (Figure 2) is a multicentre, multinational, prospective, randomised (1:1), placebo-controlled, double-blind, parallel-group clinical study. The duration of the primary assessment treatment period is 48 weeks. Approximately 200 patients with active SSc at risk of progression will enter the study. Patients are being recruited from 180 sites across 38 countries.

Oral avenciguat at a target dose of 3 mg three times daily (TID) is being compared to placebo in conjunction with local standard-of-care therapy, which may include mycophenolate mofetil (MMF), methotrexate, azathioprine and other treatments. The treatment period includes a 4-week up-titration of avenciguat from 1 to 3 mg TID, as tolerated by the patient. Avenciguat 1 mg is being given TID for 2 weeks; if tolerated, avenciguat 2 mg TID will be given for 2 weeks and then escalated to 3 mg TID. Every dose adjustment will require patients to visit the study site.

### Inclusion criteria

**Figure 3.**
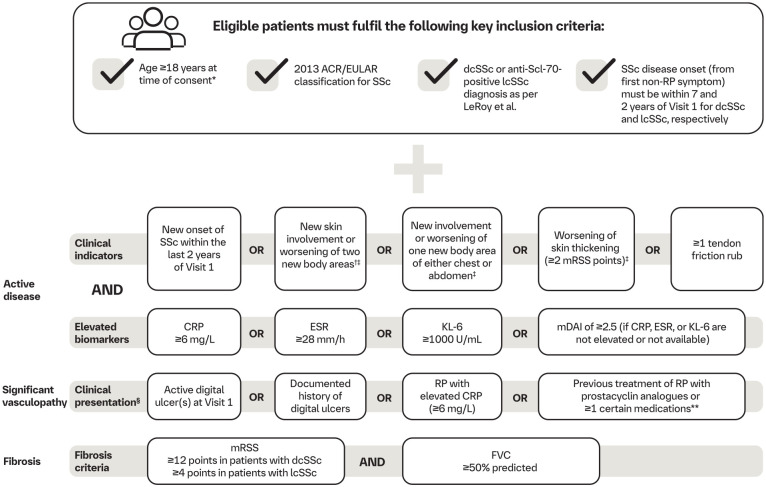
VITALISScE™ inclusion criteria. ACR: American College of Rheumatology; CRP: C-reactive protein; dcSSc: diffuse cutaneous systemic sclerosis; ESR: erythrocyte sedimentation rate; EULAR: European League Against Rheumatism; FVC: forced vital capacity; ILD: interstitial lung disease; KL-6: Krebs von den Lungen-6; lcSSc: limited cutaneous systemic sclerosis; mDAI: modified Disease Activity Index; mRSS: modified Rodnan skin score; NO: nitric oxide; PDE: phosphodiesterase; RP: Raynaud’s phenomenon; SSc: systemic sclerosis. *Or above legal age. ^†^Out of the 17 body areas defined by mRSS assessment, documented in clinical files. ^‡^Within 6 months of Visit 1. ^§^If none of the four vasculopathy criteria are met, patients can be enrolled if an ILD diagnosis by high-resolution computed tomography is confirmed. **Other medications include calcium channel blockers, nitrates, NO donors in any form (including topical), PDE5 inhibitors (including sildenafil, tadalafil, vardenafil) and non-specific PDE5 inhibitors (theophylline, dipyridamole).

Figure 3 provides an overview of the inclusion criteria used to assess eligibility for the VITALISScE™ study. More detailed inclusion and exclusion criteria can be found in Supplementary Table 1. Patients with active SSc at risk of progression who fulfil the American College of Rheumatology/European League Against Rheumatism 2013 criteria^32^ are eligible for inclusion in the study. The inclusion criteria are designed to include patients with active disease at risk of progression. For example, the study includes patients with elevated levels of blood biomarkers associated with active inflammation, such as C-reactive protein (CRP) or erythrocyte sedimentation rate (ESR), or with elevations in Krebs von den Lungen-6 (KL-6) levels, a biomarker of active, progressive interstitial lung disease (ILD).^33–35^ Alternatively, patients with an elevated modified Disease Activity Index (mDAI) score of ⩾ 2.5, which indicates active disease, are eligible for inclusion. Further information on the mDAI criteria is presented in Supplementary Table 2.

Patients must be diagnosed with limited or diffuse cutaneous SSc (lcSSc or dcSSc), as defined by LeRoy et al.^36^ Patients with dcSSc must have disease onset within 7 years of their first non-Raynaud’s phenomenon (RP) symptom. Patients with lcSSc may be included if they are anti-topoisomerase I (anti-Scl-70) antibody positive and their disease onset (defined by first non-RP symptom) is within 2 years of Visit 1. Eligible patients should also have significant vascular manifestations, i.e. the presence of active digital ulcers (DUs), a documented history of DUs, previous treatment of RP with prostacyclin analogues or ⩾1 other medication, or RP with elevated CRP. However, for patients without one of these vascular criteria, they can be enrolled if they have SSc and a confirmed diagnosis of ILD by high-resolution computed tomography.

Patients on immunosuppressive agents (MMF/sodium, methotrexate or azathioprine) will be allowed to continue these treatments if they are on a stable dose for at least 4 months prior to randomisation. If these agents have been used before, but patients are not currently on a stable dose, they should be stopped at least 4 weeks prior to randomisation. For patients on methotrexate, folic acid supplementation according to the local standard of care should be taken continuously to minimise methotrexate-associated toxicity. Patients receiving oral corticosteroids (⩽10 mg/day of prednisone or equivalent), non-steroidal anti-inflammatory drugs, angiotensin-converting enzyme inhibitors, calcium channel blockers and endothelin receptor antagonists will be allowed to continue these treatments if they are on a stable dose for at least 2 weeks prior to randomisation. Prior to randomisation, other immunomodulating/immunosuppressive treatments and corticosteroids including anakinra (1 week prior to randomisation), etanercept (2 weeks prior to randomisation), rituximab (6 months prior to randomisation), infliximab, certolizumab, golimumab, adalimumab, abatacept, tocilizumab, brodalumab and leflunomide (8 weeks prior to randomisation) should be stopped. Based on in vitro data, avenciguat is an inactivator of CYP3A4 and CYP2C8. At the maximal TID dose planned in this study, a potential drug–drug interaction is predicted between avenciguat and CYP3A4; however, a drug–drug interaction is unlikely between avenciguat and CYP2C8.

### Endpoints

Table 1 provides an overview of the endpoints to be examined in the VITALISScE™ study. The primary endpoint is the rate of decline in forced vital capacity (FVC) (mL) over 48 weeks, a well-established measure of ILD progression.^10,37^ Key secondary endpoints include absolute change from baseline in modified Rodnan skin score (mRSS) at Week 48 (patients with dcSSc only), proportion of responders based on the revised Composite Response Index in Systemic Sclerosis (rCRISS) score at Week 48 (patients with dcSSc only) and the absolute change from baseline in Health Assessment Questionnaire Disability Index score at Week 48. Additional endpoints will evaluate pharmacokinetics, safety, and biomarkers associated with SSc and the mechanism of action of avenciguat (Supplementary Table 3). Furthermore, in a magnetic resonance imaging (MRI) sub-study, changes in the blood flow of digital arteries from baseline will be evaluated up to Week 48 using a novel MRI-based score (Digital Arterial Volume Index, or DAVIX^©^).^38^

**Table 1.** Endpoints to be assessed in the VITALISScE™ study.

Endpoint	Definition
Primary	The rate of decline in FVC (mL) over 48 weeks
Key secondary	○ Absolute change from baseline in mRSS at 48 weeks in patients with dcSSc ○ The proportion of responders in patients with dcSSc based on the revised CRISS score* at 48 weeks ○ Absolute change from baseline in HAQ-DI score at Week 48
Other secondary	○ ACR-CRISS score at Week 48 in patients with dcSSc ○ Absolute change from baseline in FVC (mL and per cent predicted) at Week 48 ○ Absolute change from baseline in the PGA VAS score at Week 48 ○ Absolute change from baseline in the CGA VAS score at Week 48 ○ Composite measure of RP activity at Week 48 ○ Absolute change from baseline in DU net burden at Week 48 ○ Time to treatment failure, defined as the time to one of the following events occurring over the 48-week and extended treatment period: death or absolute decline in per cent predicted FVC ⩾ 10% relative to baseline or ⩾ 25% increase in mRSS and an increase in mRSS of > 5 points or initiation or dose change of immunomodulating/immunosuppressive therapy for clinically significant deterioration of SSc ○ Time to mRSS progression (⩾ 25% increase in mRSS and an increase in mRSS of > 5 points) in patients with dcSSc ○ Proportion of patients with dcSSc with mRSS progression (25% increase in mRSS and an increase in mRSS of > 5 points)

ACR: American College of Rheumatology; CGA: Clinician Global Assessment; CRISS: Composite Response Index in Systemic Sclerosis; dcSSc: diffuse cutaneous systemic sclerosis; DU: digital ulcer; FVC: forced vital capacity; HAQ-DI: Health Assessment Questionnaire Disability Index; mRSS: modified Rodnan skin score; PGA: Patient Global Assessment; RP: Raynaud’s phenomenon; SSc: systemic sclerosis; VAS: visual analogue scale.

* Achievement of ⩾ 20% improvement from baseline to Week 48 in ⩾ 3 of the 5 core set measures, except ⩾ 5% in per cent predicted FVC.

### Statistics

The primary and key secondary endpoints are under type-1 error control, where formal testing of the key secondary endpoints will occur only after significance is achieved for the primary endpoint. All secondary and further analyses are considered exploratory. Restricted maximum likelihood estimation, based on a random slope and intercept model, will be used to compare the adjusted rate of decline in FVC between treatment groups. This model will include fixed effects for time, treatment, baseline FVC, MMF use at baseline, anti-Scl-70 antibody status, history of/current DUs versus no history of/current DUs at baseline, presence or absence of ILD, as well as treatment-by-time and baseline-by-time interactions. Random effects for time and intercept will be included for each patient.

## Results

The VITALISScE™ study is currently ongoing. Patients are currently being enrolled and the estimated study completion date is the end of 2025. Recruitment is currently ongoing.

## Discussion

SSc is a complex, heterogeneous disease with a high unmet need for therapies that address all three aspects of disease pathophysiology: microvasculopathy, immune dysregulation and fibrosis.^5,6,24,39–41^

The sGC pathway is a promising target for the treatment of vasculopathies, as well as inflammatory and fibrotic disorders.^13,15,42,43^ Indeed, evidence from preclinical studies of previous sGC stimulators supports their potential benefits. For example, stimulation of the sGC pathway by IW-1973 or praliciguat exerted anti-inflammatory effects in animal models of hypertension, inflammation, kidney disease and non-alcoholic steatohepatitis.^27,44^ Furthermore, in mouse models of sickle cell disease, the sGC stimulator olinciguat attenuated increases in plasma biomarkers of endothelial cell activation (soluble P-selectin, soluble E-selectin and soluble intracellular adhesion molecule 1), leukocyte–endothelial cell interactions and inflammation (serum amyloid P component, serum amyloid A, plasminogen activator inhibitor-1, interleukin-6 and interleukin-1β).^45^ Finally, sGC stimulators, such as riociguat, have shown anti-inflammatory and antifibrotic effects in animal models and in vitro studies mediated by attenuation of TGF-β1 signalling, including a preclinical model of SSc.^13,15,22,23,25^

Pharmacological modulation of sGC with riociguat has been shown to exert a trend towards clinical efficacy in patients with early dcSSc and a high risk of skin fibrosis progression.^28^ In a randomised, double-blind, placebo-controlled, Phase IIb study (RISE-SSc), riociguat was well tolerated and showed potential efficacy signals in secondary and exploratory analyses. Although the primary endpoint of change in mRSS after 52 weeks was not met at the predefined significance level of p < 0.05, a prespecified responder analysis of mRSS showed a benefit for study patients receiving riociguat (p = 0.02).^28^ In addition, assessment of the decline in FVC failed to show a benefit in all patients, but did show a benefit for those with underlying ILD (–2.7 vs –7.6 in per cent predicted FVC).^28^ In the open-label long-term extension study following RISE-SSc, patients in the placebo group were switched to riociguat (2.5 mg TID) and those originally assigned to riociguat continued treatment with riociguat. Patients who switched from placebo to riociguat showed an improvement in mRSS (–2.6; 95% confidence interval –4.4 to –0.9), indicative of a potential efficacy signal.^46^

One possible reason for the attenuated effect of riociguat may be that sGC stimulators rely on haem-bound NO to modulate the cGMP pathway.^19^ Under hypoxic conditions or oxidative stress, such as in the skin and other fibrotic tissues of patients with SSc, their efficacy may be lower, leading to a reduced cGMP pathway response.^19,24^ In contrast, sGC activators directly bind to the haem site, independently of haem and NO, thereby stabilising sGC in an active form, allowing cGMP production, even in environments of hypoxia and oxidative stress.^19^

The VITALISScE™ study is the first study to assess the efficacy and safety of an sGC activator, in patients with active SSc at risk of progression. VITALISScE™ includes patients with elevated levels of CRP, ESR or KL-6, indicating active inflammation or progressive ILD.^33–35^ By including these criteria, the VITALISScE™ study is enriched for patients who are potentially at risk for disease progression.

Avenciguat has a proposed mechanism of action that is expected to be distinct from current standard-of-care therapies and sGC stimulators. In preclinical studies, avenciguat has shown in vivo modulation of vascular, autoimmune and fibrotic pathways, and may thus offer the potential to be a disease-modifying treatment addressing all three aspects of SSc pathophysiology (microvasculopathy, immune dysregulation and fibrosis).^24^ In microvascular endothelial cells, avenciguat reduced the production of the profibrotic cytokine TGF-β2 under hypoxic conditions, highlighting its potential advantages compared with sGC stimulators, such as riociguat, in environments of hypoxia and oxidative stress.^24^

The VITALISScE™ study is using an innovative method to recruit a population of patients with active SSc at risk of progression and significant vasculopathy. This involves a requirement for either elevated levels of biomarkers (CRP, ESR or KL-6) or an mDAI score ⩾ 2.5. Compared with previous trials, the prospective use of KL-6 as an entry requirement is a unique feature of this trial, which is supported by retrospective analysis of previous studies that showed KL-6 as a predictive marker of progressive lung disease.^33,47,48^

Another distinguishing feature of this trial is the use of the mDAI as an alternative to biomarkers to prospectively select patients with active disease. The mDAI score is a slightly modified version of the European Scleroderma Trials and Research group (EUSTAR) DAI score that has previously been shown to retrospectively identify patients with active disease.^49^ Both the EUSTAR DAI and mDAI assess disease activity based on tendon friction rubs, DUs, skin thickness (mRSS ⩾ 18), diffusing capacity of the lung for carbon monoxide (< 70% predicted) and elevated CRP levels. However, in contrast to the EUSTAR DAI, the mDAI includes a lower threshold of CRP (> 6 mg/L vs > 10 mg/L). In addition, EUSTAR includes a score for skin worsening assessed by the patient in the previous month, which was excluded from the mDAI. In both the original and modified DAI, a threshold of 2.5 was used to identify patients with active disease. Full details on these two criteria are shown in Supplementary Table 2. If CRP, ESR or KL-6 biomarker levels are not elevated, or test results are unavailable, patients can still be eligible for inclusion in the study if they have an mDAI score of ⩾ 2.5. Finally, we have developed a definition of significant vasculopathy in order to enrich the study population with patients who have DUs, a history of DUs or who are at significant risk of DUs.

### Conclusions

The VITALISScE™ study is the first trial to evaluate the efficacy, safety and tolerability of an sGC activator in patients with active SSc at risk of progression. Building on promising preclinical data for avenciguat, VITALISScE™ will assess its potential to address all three aspects of SSc pathogenesis, namely microvasculopathy, immune dysregulation and fibrosis. The study uses a novel approach to patient selection to enrich the study population with patients who have active SSc at risk of progression and significant vasculopathy.

## Supplemental Material

sj-pdf-1-jso-10.1177_23971983241291923 – Supplemental material for A Phase II study of avenciguat, a novel soluble guanylate cyclase activator, in patients with systemic sclerosis: Study design and rationale of the VITALISScE™ study Supplemental material, sj-pdf-1-jso-10.1177_23971983241291923 for A Phase II study of avenciguat, a novel soluble guanylate cyclase activator, in patients with systemic sclerosis: Study design and rationale of the VITALISScE™ study by Dinesh Khanna, Jeska de Vries-Bouwstra, Anna-Maria Hoffmann-Vold, Masataka Kuwana, Andrea Hsiu Ling Low, Susanna Proudman, Mary Flack, Anjli Kukreja, Nora Fagan and Oliver Distler in Journal of Scleroderma and Related Disorders
