# Supplementary material for: A Phase II study of avenciguat, a novel soluble guanylate cyclase activator, in patients with systemic sclerosis: Study design and rationale of the VITALISScE™ study
Source: J Scleroderma Relat Disord. 2024 Nov 7;10(1):27–35. doi: 10.1177/23971983241291923 (PMC11559521; doi:10.1177/23971983241291923)
Supplement: sj-pdf-1-jso-10.1177_23971983241291923 – Supplemental material for A Phase II study of avenciguat, a novel soluble guanylate cyclase activator, in patients with systemic sclerosis: Study design and rationale of the VITALISScE™ study [file sj-pdf-1-jso-10.1177_23971983241291923.pdf]

Supplement

Supplementary Table 1: Inclusion and exclusion criteria for the VITALISScE™ study

Inclusion criteria

- Male or female patients aged ≥18 years (or above legal age in respective country) at the time of consent
- Patients must fulfil the 2013 ACR/EULAR classification criteria for SSc[1]
- Clinical diagnosis of lcSSc or dcSSc as defined by LeRoy et al. 1988;[2] patients with lcSSc may be included if they are anti-Scl-70-antibody positive
- dcSSc disease onset, defined by first non-RP symptom, must be within 7 years of Visit 1; lcSSc disease onset must be within 2 years of Visit 1
- Active disease (new onset of SSc ≤2 years prior to Visit 1 **or** new skin involvement/worsening of two new body areas within 6 months of Visit 1 [out of the 17 body areas defined by the mRSS] **or** new involvement/worsening of one new body area if either the chest or abdomen within 6 months of Visit 1 **or** worsening of skin thickening (≥2 mRSS points) within 6 months of Visit 1 **or** ≥1 tendon friction rub)
- Elevated biomarkers (CRP ≥6 mg/dL **or** erythrocyte sedimentation rate ≥28 mm/h **or** KL-6 ≥1000 U/mL) on Visit 1
    - If none of these are met, the patient can be entered into the study if the modified EUSTAR DAI is ≥2.5, as previously described.[3] The criteria for the modified EUSTAR DAI can be found in Supplementary Table 2
- Evidence of significant vasculopathy defined as active DU on Visit 1 **or** a documented history of DUs **or** a previous treatment for RP (with prostacyclin analogues or ≥1 other medication including calcium channel blockers, nitrates, NO donors in any form, including topical; PDE5 inhibitors, non-specific PDE inhibitors) or RP with elevated CRP ≥6 mg/L
    - If none of these criteria are met, the patient can be entered into the study if a diagnosis of ILD has been confirmed
- Evidence of fibrosis at Visit 1, defined as mRSS of ≥12 points in patients with dcSSc/mRSS of ≥4 points in patients with lcSSc **and** FVC ≥50% of predicted normal

Exclusion criteria

- Any known form of pulmonary hypertension
- Pulmonary disease with FVC <50% of predicted at screening
- Other autoimmune connective tissue diseases, except for fibromyalgia, scleroderma-associated myopathy and secondary Sjögren's syndrome
- DLco (haemoglobin corrected) <40% of predicted at screening

- Any history of scleroderma renal crisis within the last 6 months
- Estimated glomerular filtration rate <30 mL/min/1.73 m^2 (Chronic Kidney Disease Epidemiology formula) or on dialysis at screening
- Cirrhosis of any Child–Pugh class (A, B or C)
- Cholestasis at present, or ALP >4 x ULN, or ALP >2 x ULN and GGT >3 x ULN at screening
- Known, severe gastric antral telangiectasias
- Any history of bronchial artery embolisation **or** massive haemoptysis (massive haemoptysis defined as acute bleeding >240 mL in a 24-hour period or recurrent bleeding >100 mL/day over consecutive days)
- Active haemoptysis or pulmonary haemorrhage, including events managed by bronchial artery embolisation
- Unstable cardiovascular, pulmonary (other than study indication) **or** other disease within 6 months prior to Visit 1 and/or during the screening period (e.g. acute coronary artery disease, heart failure or pulmonary embolism)
- Systolic blood pressure <100 mm Hg **or** known history of moderate or severe symptomatic orthostatic dysregulation as judged by the Investigator before start of study treatment
- Sitting heart rate <50 beats per minute at the screening visit
- Laboratory values: haemoglobin <9.0 g/dL, WBC count <3000/mm^3 ($<3 \times 10^9$/L), platelet count <100,000/mm^3 ($<100 \times 10^9$/L)
- Known heart failure with left ventricular ejection fraction <40% prior to screening
- Marked baseline prolongation of QT/QT corrected for heart rate by Fridericia's cube root formula interval (by a repeated demonstration in at least 2 ECG measurements within the triplicate or in two triplicates of a QTcF interval (>450 ms in male and >470 ms in female patients) or risk factors for Torsades de Pointes (for example, heart failure, hypokalaemia, family history of Long QT Syndrome)
- Use of the following treatments and therapies:
  - Nitrates or NO donors (e.g. amyl nitrate) in any form, including topical PDE5 inhibitors (e.g. sildenafil, tadalafil, vardenafil); and nonspecific PDE inhibitors (theophylline, dipyridamole) within 2 weeks prior to randomisation
  - Prostacyclin analogues (oral beraprost for DUs/RP and short-term/intermittent therapy of up to 21 days with intravenous prostacyclin

analogues for digital/vascular lesions are allowed) within 2 weeks prior to randomisation

- Nintedanib, pirfenidone, terguride, tyrosine-kinase inhibitors (e.g. imatinib, nilotinib, dasatinib), Janus kinase inhibitors within 2 weeks prior to randomisation
- sGC stimulators/activators (other than avenciguat) within 4 weeks prior to randomisation
- Treatment with clinically relevant OATP1B1/3 inhibitors and clinically relevant UGT inhibitors/inducers within 4 weeks prior to randomisation
- Drugs with known risk of Torsade de Pointes within 5 half-lives prior to randomisation
- Other investigational drugs within 1 month or 5 half-lives (whichever is greater) prior to randomisation
- Ultraviolet phototherapy within 6 weeks prior to randomisation

o Use of the following immunomodulating/immunosuppressive treatments and corticosteroids:

- Anakinra within 1 week prior to randomisation
- Etanercept within 2 weeks prior to randomisation
- Cyclophosphamide, cyclosporine A, tacrolimus, sirolimus, colchicine, D-penicillamine, mizoribine and intravenous immunoglobulin within 4 weeks prior to randomisation
- Hydroxychloroquine within 6.5 months (5 half-lives) prior to randomisation
- Infliximab, certolizumab, golimumab, adalimumab, abatacept, tocilizumab, brodalumab and leflunomide within 8 weeks prior to randomisation
- Rituximab or other anti-CD20 antibodies within 6 months prior to randomisation
- Non-investigational or investigational cell-depleting therapies, including but not limited to alemtuzumab, anti-CD4, anti-CD5, anti-CD3 and anti-CD19 within 18 months prior to randomisation
- Previous treatment with chlorambucil, bone marrow transplantation, total lymphoid irradiation, thalidomide, antithymocyte globulin, plasmapheresis or extracorporeal photopheresis
- Oral prednisone >10 mg/day or equivalent, intravenous and intramuscular corticosteroids within 2 weeks prior to randomisation

- Local background standard of care must not be terminated for the patient to be eligible to participate in the study. Patients with SSc-ILD who, in the opinion of the Investigator, require approved treatments that are not allowed in this study (where such therapy is available and considered standard of care) should not be included in this study
- Relevant chronic or acute infections including but not limited to human immunodeficiency virus and viral hepatitis. The corresponding laboratory tests will be performed during screening. A patient can be re-screened if the patient was treated and is cured from the acute infection
- The patient has an active infection with SARS-CoV-2 (or is known to have a positive test) from screening until randomisation
- Major surgery (major according to the Investigator's assessment) planned during the study
- Any documented active or suspected malignancy or history of malignancy within 5 years prior to screening, except appropriately treated basal or squamous cell carcinoma of the skin or in situ carcinoma of uterine cervix
- History of clinically relevant allergy/hypersensitivity that would interfere with study participation including allergy to investigational product/placebo or its excipients
- Any other medical condition that in the Investigator's opinion poses a safety risk for the patient or may interfere with the study objectives
- Patients not expected to comply with the protocol requirements or not expected to complete the study as scheduled (e.g. chronic alcohol or drug abuse or any other condition that, in the Investigator's opinion, makes the patient an unreliable study participant)
- Previous randomisation/treatment in this study
- Currently enrolled in another investigational device or drug study, or less than 1 month or 5 half-lives (whichever is greater) since ending another investigational device or drug study(s) or receiving other investigational treatment(s) prior to randomisation
- Women who are pregnant, nursing or who plan to become pregnant while in the study
- MRA sub-study: Contraindication to MRI or inability to undergo MRI (e.g. implanted medical devices that are contraindicated for MRI and cannot be removed (e.g. cardiac pacemaker, neurostimulation systems), severe claustrophobia)
- Patients who are legally institutionalised according to national law.

ACR, American College of Rheumatology; ALP, alkaline phosphatase; CD, cluster of differentiation; CRP, C-reactive protein; dcSSc, diffuse cutaneous systemic sclerosis; DLco, diffusing capacity of the lung for carbon monoxide; DU, digital ulcer; ECG, electrocardiogram; EULAR, European League Against Rheumatism; EUSTAR DAI, European Scleroderma Trials and Research group Disease Activity Index; FVC, forced vital capacity; GGT, gamma-glutamyl transpeptidase; ILD, interstitial lung disease; KL-6, Krebs von den Lungen 6; lcSSc, limited cutaneous systemic sclerosis; MRA, magnetic resonance angiography; MRI, magnetic resonance imaging; mRSS, modified Rodnan skin score; NO, nitric oxide; PDE, phosphodiesterase; QTcF, QT corrected for heart rate by Fridericia's cube root formula; RP, Raynaud's phenomenon; sGC, soluble guanylate cyclase; SSc, systemic sclerosis; UGT, Uridine 5'-diphospho-glucuronosyltransferase; ULN, upper limit of normal; WBC, white blood cell.

Supplementary Table 2: Criteria for EUSTAR DAI and modified EUSTAR DAI[3,4]

**Category**	**EUSTAR DAI score**	**mDAI score**
Skin worsening in previous month	1.5	NA
Tendon friction rubs	2.25	2.25
High-sensitivity CRP	>10 mg/L = 2.25	>6 mg/L = 2.25
Digital ulcers	1.5	1.5
mRSS ≥18<br>mRSS <18	1.5<br>mRSS x 0.084	1.5<br>mRSS x 0.084
Dlco <70% of predicted value	1.0	1.0

CRP, C-reactive protein; Dlco, diffusing capacity of the lung for carbon monoxide; EUSTAR, European Scleroderma Trials and Research group Disease Activity Index; mDAI, modified Disease Activity Index; mRSS, modified Rodnan skin score; NA, not assessed.

Supplementary Table 3: Additional endpoints

Category	Endpoints
Efficacy	• Absolute change from baseline in the Functional Assessment of Chronic Illness Therapy-Fatigue Scale score at Week 48<br>• Absolute change from baseline in Scleroderma Skin Patient Reported Outcome at Week 48<br>• Absolute change from baseline in European Quality of Life 5 Dimension at Week 48<br>• Absolute change from baseline in Worst Pain Numeric Rating Scale at Week 48<br>• Absolute change from baseline in the six individual Scleroderma Health Assessment Questionnaire domains (pain, intestinal problems, respiratory problems, RP, finger ulcers, disease severity) at Week 48<br>• Patient's Global Impression of Change scale at Week 48<br>• Absolute change from baseline in per cent predicted DLco at Week 48<br>• Global Rank Composite Score at the end of the 48-week primary assessment treatment period for patients who do not participate in the extended treatment period (or at the end of the extended treatment period)<br>• Proportion of patients who have treatment failure or discontinue treatment over the 48-week or extended treatment period<br>• Annual rate of FVC decline over the primary assessment period and extended treatment period<br>• Change from baseline in presence or absence of tendon friction rubs at Week 48<br>• Change from baseline in joint involvement (tender and swollen joint count –28) at Week 48<br>• Absolute change from baseline in RCS at Week 48<br>• Absolute change from baseline in mRSS at Week 48<br>• Proportion of responders based on the revised CRISS at Week 48 (achievement of ≥20% improvement from baseline to Week 48 in at least 3 of the 5 core set measures, except ≥5% in per cent predicted FVC)<br>• Proportion of responders in patients with dcSSc based on the revised

Category	Endpoints
	CRISS at Week 48 (improvement from baseline to Week 48 in at least 2 of the 5 core set measures [≥25% decrease for mRSS, HAQ-DI, PGA, CGA and/or ≥5% increase for per cent predicted FVC], with worsening in no more than one component [≥25% increase for mRSS, HAQ-DI, PGA, CGA and/or ≥5% decrease for per cent predicted FVC])<br>• Proportion of responders based on the revised CRISS at Week 48 (improvement from baseline to Week 48 in at least 2 of the 5 core set measures [≥25% decrease for mRSS, HAQ-DI, PGA, CGA and/or ≥5% increase for per cent predicted FVC], with worsening in no more than one component [≥25% increase for mRSS, HAQ-DI, PGA, CGA and/or ≥5% decrease for per cent predicted FVC])
Pharmacokinetics	• Further PK parameters will be calculated through Week 36 if feasible. These PK parameters may include, but are not limited to:<br>o C_{max} (maximum measured concentration of the analyte [avenciguat] in plasma)<br>o t_{max} (time from dosing to maximum measured concentration of the analyte in plasma)<br>o AUC_{t1-t2} (area under the concentration–time curve of the analyte in plasma over the time interval t1 to t2)
Biomarkers	• Disease- and pathway-related biomarkers to be examined include, but are not limited to:<br>o KL-6 and CRP<br>• A sub-study will look at the change in Digital Artery Volume Index (DAVIX©), a novel quantitative magnetic resonance imaging-based score for the assessment of the blood flow in the arteries, from baseline up to Week 48
Safety	• At set points in the study, during a clinic visit, patients will receive a physical examination, vital signs examination, laboratory parameters and electrocardiograms*<br>• AEs and SAEs will be collected and documented during the course of the study. An SAE is defined as any AE that results in death, is life-threatening, requires hospitalisation or prolongation of existing

Category	Endpoints
	hospitalisation, results in persistent or significant disability or incapacity, is a congenital anomaly or birth defect, or is deemed serious for any other reason<br>• All AEs will be coded using the Medical Dictionary for Drug Regulatory Activities. All treated patients will be included in the safety analysis. This will be descriptive in nature

*Electrocardiograms will be performed in triplicate.

AE, adverse event; CGA, Clinician Global Assessment; CRISS, Composite Response Index in Systemic Sclerosis; CRP, C-reactive protein; dcSSc, diffuse cutaneous systemic sclerosis; DLco, diffusing capacity of the lung for carbon monoxide; FVC, forced vital capacity; HAQ-DI, Health Assessment Questionnaire Disability Index; KL-6, Krebs von den Lungen 6; mRSS, modified Rodnan skin score; PGA, Patient Global Assessment; PK, pharmacokinetic; RCS, Raynaud's Condition Score; RP, Raynaud's phenomenon; SAE, serious adverse event.

References

1. van den Hoogen F, Khanna D, Fransen J, et al. 2013 classification criteria for systemic sclerosis: an American College of Rheumatology/European League against Rheumatism collaborative initiative. *Arthritis Rheum* 2013; 65: 2737-2747.
2. LeRoy EC, Black C, Fleischmajer R, et al. Scleroderma (systemic sclerosis): classification, subsets and pathogenesis. *J Rheumatol* 1988; 15: 202-205.
3. Herrick A, Pope J, Carreira P, et al. Effects of nintedanib in patients with systemic sclerosis-associated interstitial lung disease (SSC-ILD) in subgroups by disease activity index. Presented at: *European Congress of Rheumatology (EULAR)*, Milan, Italy, 31 May-3 June 2023.
4. Valentini G, Iudici M, Walker UA, et al. The European Scleroderma Trials and Research group (EUSTAR) task force for the development of revised activity criteria for systemic sclerosis: derivation and validation of a preliminarily revised EUSTAR activity index. *Ann Rheum Dis* 2017; 76: 270-276.
